# Earthquake-induced structural deformations enhance long-term solute fluxes from active volcanic systems

**DOI:** 10.1038/s41598-018-32735-1

**Published:** 2018-10-04

**Authors:** Takahiro Hosono, Jens Hartmann, Pascale Louvat, Thorben Amann, Kirstin E. Washington, A. Joshua West, Koki Okamura, Michael E. Böttcher, Jérôme Gaillardet

**Affiliations:** 1 Priority Organization for Innovation and Excellence, Kumamoto University, 2-39-1 Kurokami, Kumamoto, 860-8555 Japan; 2 Department of Earth and Environmental Science, Kumamoto University, 2-39-1 Kurokami, Kumamoto, 860-8555 Japan; 3 Institut de Physique du Globe de Paris, Sorbonne Paris Cité, Univ Paris Diderot, UMR, 7154 CNRS Paris, France; 4 Institute for Geology, Universität Hamburg, Center for Earth System Research and Sustainability (CEN), Bundesstrasse 55, 20146 Hamburg, Germany; 5 Department of Earth Sciences, University of Southern California, 3651 Trousdale Parkway, Los Angeles, CA 90089 USA; 6 Geochemistry & Isotope Biogeochemistry Group, Leibniz Institute for Baltic Sea Research (IOW), Seestrasse 15, D-18119 Warnemünde, Germany

## Abstract

Evidence for relationships between seismotectonic activity and dissolved weathering fluxes remains limited. Motivated by the occurrence of new springs emerging after the 2016 Kumamoto earthquake and supported by historical groundwater data, this study focuses on the long-term effect of near-surface structural deformation on the contribution of deep, highly saline fluids to the solute fluxes from the Aso caldera, Kyushu, Japan. Available hydrologic and structural data suggest that concentrated, over-pressured groundwaters migrate to the surface when new hydraulic pathways open during seismic deformation. These new springs have a hydrochemical fingerprint (including δD_H2O_, δ^18^O_H2O_, δ^7^Li, δ^11^B, δ^18^O_SO4_, and δ^34^S_SO4_) indistinguishable from long-established confined groundwater that likely reflects a mixture of infiltrated meteoric water with high-sulfate hydrothermal fluids. A comparison of historical hydrochemistry data and patterns of past seismicity suggests that discharge of deep fluids is associated with similar deformation structures to those observed during the Kumamoto earthquake, and that seismic activity plays an important role over historic timescales in delivering the majority of the solutes to the caldera outlet, sustaining fluxes that are amongst the world’s highest. This upwelling mechanism might be relevant for other systems too, and could contribute to the over-proportional share of active volcanic areas in global weathering fluxes.

## Introduction

Active volcanic areas contribute a disproportionately large component to the global flux of dissolved elements delivered from the land to the oceans, both in comparison to other lithologies^1,2^ and to inactive volcanic systems^3^. Several processes may cause enhanced solute fluxes from active volcanic areas, including the reaction of ecosystem-recycled CO_2_ with relatively young fresh mineral material^4–6^, volcanic acid mediated rock alteration in geothermal fields^7–12^, and deep, solute-rich crustal fluids released to the surface through crustal pathways^13–16^. These processes have distinct implications for weathering budgets and particularly for the long-term global carbon cycle. However, the distribution, source, and discharge mechanism of deep fluids, and their impact on watershed hydrochemistry and long-term lateral weathering fluxes, are not well understood in the global context because of limited study opportunities. Earthquakes can trigger an increase in dissolved geochemical fluxes^13–20^ partly by opening new pathways for deep fluid discharge to the surface^13–16^. Even though active volcanism is sometimes accompanied by seismotectonic processes that may systematically facilitate the release of solute-rich deep fluids^21,22^, the role of seismotectonic processes in sustaining the high apparent weathering fluxes from volcanic systems is not known.

**Figure 1:**
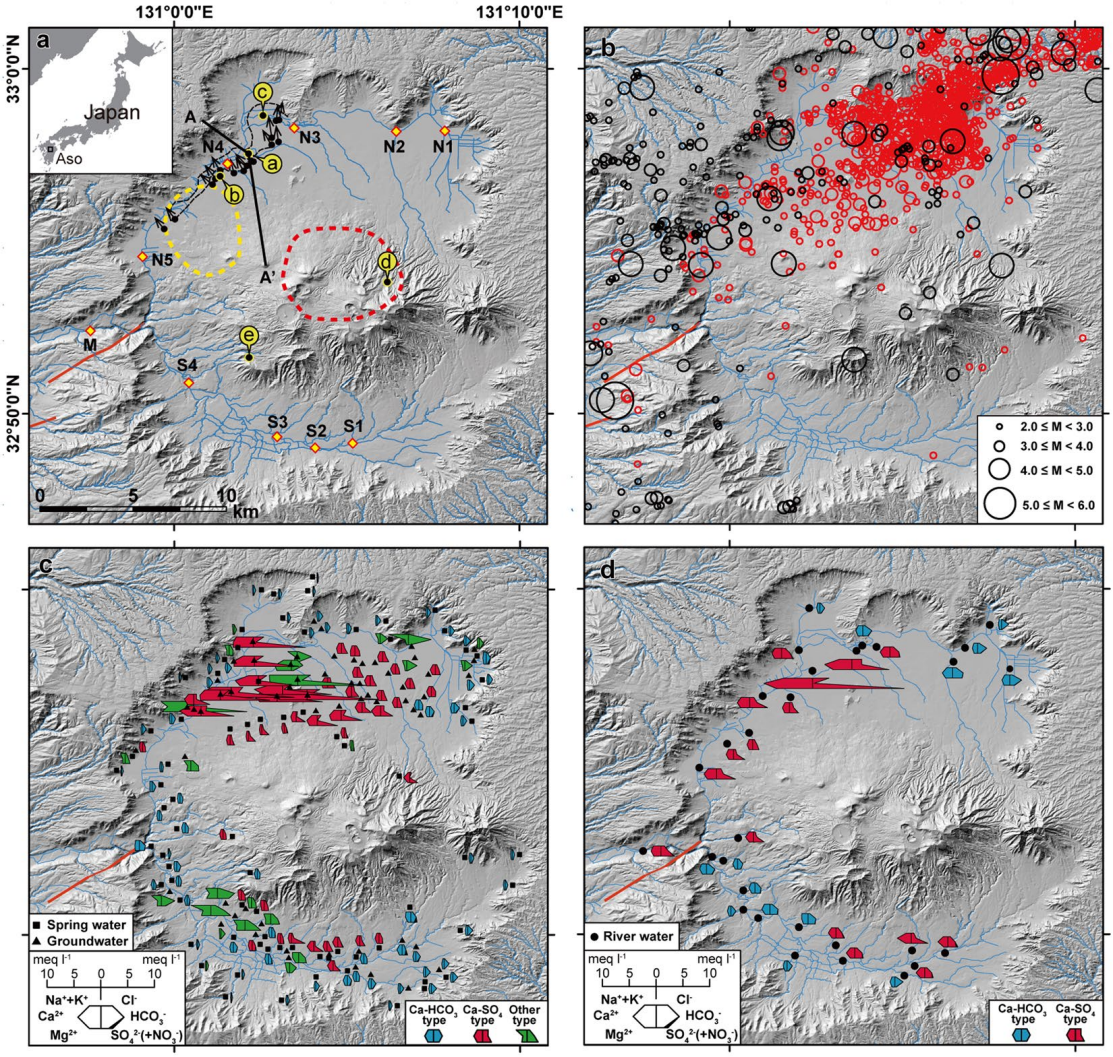
Seismotectonic and hydrochemistry maps of Aso caldera watershed. Spatial distribution of (**a**) sampling stations and dominant extensional fissures (black dots with arrows), and associated horizontal land sliding (black dotted line area) observed after the 2016 Kumamoto earthquake^27–29^, (**b**) earthquake epicenters before (1923~: black circle) and after the 2016 main shock (red circle), and water hydrochemistry for (**c**) spring water and groundwater and (**d**) rivers based on data from 1968–1995 (Supplementary Table 1). Samples for volcano-hydrothermal fluids (labeled a–e in yellow circles of Fig. 1a; see Table 1 for details) and river waters (diamond, N1 to N5, S1 to S4 and M) in Fig. 1a correspond to the samples in Table 1 and Fig. 4, respectively. Locations of reported low resistivity zone^31^ for hypothesized melt finger in the deep crust (9.0–10.0 km in depth) and magma chambers beneath central volcanoes (2.0–2.5 km in depth) are shown as yellow dotted line and red dotted line, respectively. Schematic cross section A-A’ is shown in Fig. 3. Major active faults of Futagawa-Hinagu fault systems are shown as red lines. The map was illustrated by using ArcGIS Desktop (Esri).

**Figure 2:**
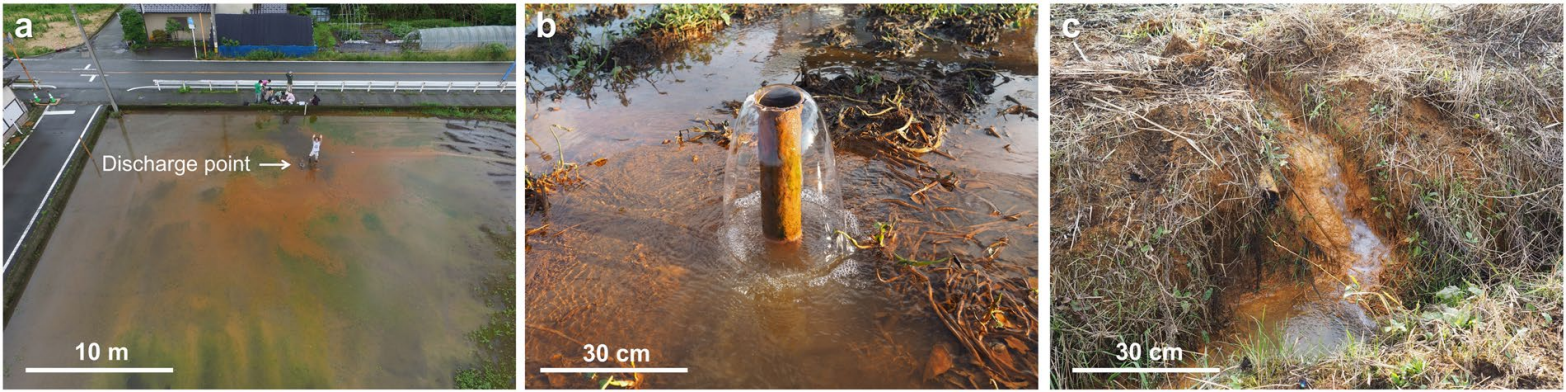
Pictures of highly saline fluid discharges in the northwestern plain area. (**a**) A wide view and (**b**) close-up picture of highly saline spring water that appeared after the 2016 Kumamoto earthquake (corresponding to ‘a’, high saline fluid after the quake in Table 1 and Fig. 1a). (**c**) Picture of highly saline spring water pre-existed before the 2016 Kumamoto earthquake (corresponding to ‘b’, high saline fluid before the quake “Kayahara” in Table 1 and Fig. 1a).

Most of the surface land of the Kyushu Islands (36,750 km^2^), in the southern part of the Japanese volcanic arc, is located within a highly active area with high weathering potential^1,2^. The Aso caldera watershed (Fig. 1) is a hot-spot of weathering in this region^6,23,24^ and is also crossed by a major tectonic line in its northwestern plain (see Methods). One of the curious hydrological phenomena after the 2016 Kumamoto earthquake (see Methods) was the appearance of new highly saline fluid discharging in this northwestern plain (Figs 1a and 2a,b, and Table 1), where the earthquake caused major surface ruptures (Fig. 1a,b). The release of solute rich water along these structures raises the hypothesis that the combination of major tectonic and volcanic activities leads to locally elevated hydrochemical fluxes in the northwestern plain of the caldera. This study considers how such structures may sustain the high solute fluxes observed in this region, and thus contribute to the high apparent weathering fluxes from active volcanic systems.

**Table 1:** A summary of hydrochemistry and isotope ratios of volcano-hydrothermal fluids within Aso caldera watershed.

	Temp	pH	TDS	Alkalinity	SO_4_^2−^	Cl^−^	Ca^2+^	Li^+^	B	δD_H2O_	δ^18^O_H2O_	δ^7^Li	δ^11^B	δ^34^S_SO4_	δ^18^O_SO4_
	°C		mmol l^−1^	mmol l^−1^	mmol l^−1^	mmol l^−1^	mmol l^−1^	μmol l^−1^	μmol l^−1^	‰	‰	‰	‰	‰	‰
a. Highly saline fluid after the quake	23.1	6.7	30.9	0.52	11.50	3.95	3.89	18.2	58.4	−56.1	−8.66	2.6	2.6	17.5	13.5
b. Highly saline fluid before the quake “Kayahara”	16.2	6.7	23.7	0.61	4.59	5.81	3.84	9.3	26.1	−54.2	−8.52	NA	1.4	10.5	9.8
c. Hot spring “Uchinomaki”	40.4	7.1	22.9	3.20	3.44	1.69	1.57	23.6	76.7	−53.8	−8.60	9.0	−2.8	17.5	12.8
d. Acidic stream	19.6	4.0	5.40	1.03	1.42	1.14	0.95	0.7	3.7	−44.0	−7.27	4.8	3.2	7.2	9.5
e. Geothermal water	40.0	2.7	7.29	ND	4.99	0.17	0.74	1.5	57.3	−53.6	−7.98	7.64	−2.4	2.6	−2.8

See Fig. 1a for the location of samples a–e.

TDS = [Alkalinity] + [SO_4_^2−^] + [Cl^−^] + [NO_3_^−^] + [Na^+^] +[K^+^] + [Ca^2+^] + [Mg^2+^].

ND: not determined.

NA: not analyzed.

The samples for three volcano hydrothermal fluids (a. high saline fluid after the quake, b. high saline fluid before the quake “Kayahara”, and c. hot spring “Uchinomaki”) were collected in July 2016 after the 2016 Kumamoto earthquake (see Methods).

Average data are shown for d. acid stream and e. geothermal water and complete dataset is supplied in Supplementary Table 2.

## Discharge mechanism and origin of the new spring

Compressional crustal strain and pressurized fluids affected by a heat source can drive co- to post-seismic upwelling of deep fluid through crustal ruptures^14,25,26^. In contrast, the new fluid source observed in the Aso caldera (a high-salinity fluid after the earthquake, Figs 1a, 2a,b, and Table 1) appeared around an open graben-like structure in the surface sedimentary deposits of the northwestern plain^27–29^, and both cold and hot artesian springs are observed in this region, suggesting heat-pressurization is not a key factor. Satellite radar interferometry images^27,28^ and direct borehole observations confirm that northward horizontal sliding of the sediment block (by more than 2 m) occurred during the 2016 Kumamoto earthquake just above a heated fluid reservoir at around 50 m depth^29^. Open fissures were formed predominantly along the eastern side of the slid block (see Fig. 1a for their locations) and reached the depth of a local confined aquifer system that had previously developed in the uppermost sediment pile^29^. We propose that this aquifer is the likely source of discharging fluids, and that release of these fluids to the surface was facilitated by the high permeability pathways that opened along the new fractures during the earthquake. Other similar new springs were also discovered in the same structural setting after the 2016 earthquake, although we sampled and analyzed only one.

**Figure 3:**
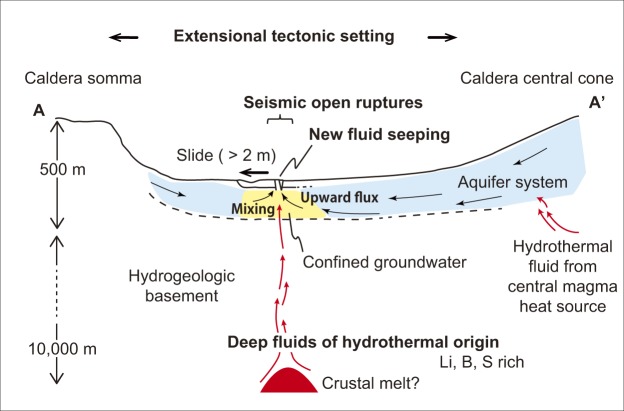
Deep fluid discharge mechanism. Schematic illustration of confined groundwater (meteoric water admixed with deep high-sulfate hydrothermal fluids) upwelling along gradients in hydraulic potential via surface ruptures (A-A’ section in Fig. 1a). The surface structural deformation and deeper melt source location are adapted from horizontal sliding model^29^ and electrical resistivity images^31^, respectively.

In general, the confined groundwater hydraulic potential in the northwestern plain is higher than the ground surface level, as evidenced by the presence of natural cold springs (e.g., Kayahara, Fig. 2c and Table 1), artesian deep groundwater, and hot springs (Uchinomaki, Table 1), all observed for several decades in this region. This structural and hydrogeological context leads us to suggest that the observed new fluids discharging after the Kumamoto earthquake migrated towards the surface from the confined groundwater by pressure release^30^ through either visible (as opening fissure) or invisible (small structural weak and conduit) pathways (Fig. 3), a mechanism also responsible for pre-existing artesian wells.

Further evidence supporting a deep aquifer origin of the post-earthquake springs comes from the water chemistry. Compared with surface waters from the Aso caldera, the new fluid is characterized by lower stable hydrogen and oxygen isotope ratios (δD_H2O_ = −56.1‰ and δ^18^O_H2O_ = −8.66‰, Table 1), similar to the composition of confined groundwater at 50–200 m depth from the surface (−58‰ < δD_H2O_ < −54‰ and −9.0‰ < δ^18^O_H2O_ < −8.5‰)^8,30^. Moreover, the new spring water and similar highly saline water from springs that pre-dated the earthquake (Kayahara) (Fig. 2) are characterized by low lithium and boron stable isotope ratios (δ^7^Li = 2.6‰ and δ^11^B = 1.4 to 2.6‰). These compositions are typical of volcano-hydrothermal fluids globally, with low apparent fractionation often attributed to high temperatures of associated water-rock reaction^9–11,15^. Together with high sulfate content and slightly elevated water temperature of the new spring (23.1 °C; Table 1 and Supplementary Table 1), these geochemical indicators are consistent with meteorically-derived groundwater admixed with deeper fluids of hydrothermal origin.

### Source of highly concentrated hydrothermal fluids contributing to springs in the northwestern plain

Identifying the source of the deep hydrothermal contributions to the solute-rich springs is important for understanding the role of these fluids in the caldera system. The hydrothermal deep fluids are likely to originate from a location near hot magma, with two probable candidates being the central volcanic cones, or melt pockets beneath the observed springs. Under the center of the volcanic cones, magma ascends from about 16 km depth toward the surface^31^. Contributions from the central magma chamber were suggested for groundwater with deep flow paths using a δ^13^C_DIC_ tracer method^8^; although this has not been considered for the waters from the northwestern plain specifically, similar waters could contribute to the springs in the region. Alternatively, a recent electrical resistivity survey^31,32^ suggests the presence of a localized melt finger deeper than 8 km beneath the area seeping deep fluids in the northwestern plain (yellow dotted line in Fig. 1a). Extensional crustal structures along the Oita-Kumamoto Tectonic Line crossing the northwestern plain^33^ could allow hydrothermal fluids originating from near this melt finger to ascend through crustal pathways and mix with upper surface aquifer systems (Fig. 3). A similar mechanism was found in southern Kyushu where fluid continued upwelling over geological time scales^34^.

Distinguishing between these two scenarios for the origin of the hydrothermal component in spring waters of the northwestern plain requires understanding the wider context of hydrochemistry across the Aso caldera. Historical hydrochemical monitoring in the region (1968–1995, see Methods) provides a picture of how subsurface and surface waters evolve geochemically along flow paths, and how this chemical evolution relates to magmatic, seismic, and tectonic activities (Fig. 1). In studied watersheds across the caldera, mainly two different water types can be distinguished in terms of major dissolved ion concentrations: Ca-HCO_3_ type and Ca-SO_4_ type (Fig. 1c,d). The watersheds of the caldera rim areas, devoid of volcano-hydrothermal activity, are mainly of the Ca-HCO_3_ type. Contrastingly, those around the central mountain and lowland areas, including the northwestern plain, have predominantly Ca-SO_4_ type waters with much higher total solute concentrations. Supply of acid from high-temperature, SO_2_-rich magmatic gases to the subsurface water body can enhance rock alteration and weathering, increasing sulfate concentrations of groundwater and adding substantial amounts of other rock-derived dissolved ions (this type of rock dissolution is described here as ‘magmatic S-mediated weathering’ and is discussed in greater detail below). The elevation in concentrations due to this process is likely to be mediated by the distance from the heat sources (Fig. 1a), the availability of high-permeability flow pathways, and the potential to mix with percolated fresher surface water^8^.

The morphology and topography of the Aso caldera watersheds are characterized by symmetrical features between the northern and southern catchments, except for a slightly larger catchment area in the north, partly associated with the relatively flat plain (Fig. 1). The geology within the watersheds is approximately symmetric in terms of lithology and general stratigraphical features, although the central mountains consist of several complex volcanic cones of different compositions (see Methods). Similar patterns in groundwater flow dynamics and hydrochemistry have also been identified in the north and south^30^, and these are partly reflected in the results from our study (Fig. 1c): subsurface waters recharged at the caldera rim and central mountains evolve to a Ca-HCO_3_ type, while Ca-SO_4_ type waters discharge into the plain areas, and are characterized by longer and deeper flow paths especially in the case of the central part of the northern plain.

It has been argued that the moderately elevated concentrations of Ca-SO_4_ type subsurface waters in the central to eastern part of the northern plain (Fig. 1c) are caused by the contribution of magmatic components originating from under the central volcanoes via deep groundwater flow paths^8,30^. This scenario is consistent with their proximity to upwelling magmas under the central mountains (Fig. 1a)^31^. Similarly, hydrothermal inputs from the central volcanoes are reflected in hydrochemical anomalies in the form of Ca-SO_4_ and Na-SO_4_ type waters in southern catchments, most prominently south of a large geothermal field (site “e” in Fig. 1a), although these chemical features are more weakly expressed at the foot of the central mountains in the south compared to the north (Fig. 1c).

In comparison to these data from around the caldera, waters in the northwestern plain, where new springs were observed following the 2016 Kumamoto earthquake, stand out as chemically distinct. Historical hydrochemical datasets in the vicinity of the new water sources (location “a” in Fig. 1a compared with Fig. 1c) suggest groundwaters with a stronger geochemical fingerprint of deep hydrothermal sources. These groundwaters are comparable in composition to the newly appeared spring that we have analyzed, with higher concentrations than reported anywhere else in the Aso caldera. Stable isotope analysis has revealed that groundwater flow patterns in this area are comparable to those in the central and eastern part of northern plain^30^, suggesting that flow-path differences (e.g., preferential advection of fluids from the central cone due to stratigraphic or permeability controls) are unlikely to explain the distinct hydrochemistry on their own. In addition, the anomalous area in the northwestern plain is on average farther from the central volcano heat sources than the location of other groundwaters that have a hydrothermal imprint, yet the waters in the northwestern plain have higher solute concentrations despite their greater distance (Fig. 1a). Together, this evidence suggests that additional heat sources outside of the central volcanic area are likely responsible for the hydrothermal contributions in the northwestern plain. As hypothesized based on an electrical resistivity survey^31^, a deep melt finger beneath this area (Fig. 1a, yellow dashed line) might be the additional source for hydrothermal influences that could explain the observed geochemical anomaly in the spring and groundwaters^32^ (Fig. 3). The slightly elevated temperature of the new observed spring (Fig. 1a, location “a”) and a hot water reservoir (40.4 °C; Uchinomaki, Table 1) at the level of the confined aquifer system (~50 m deep)^29^ support this scenario.

## Spatiotemporal relationship between hydrochemistry and seismotectonic activity

The hydrochemical anomaly in the northwestern plain, attributed here to contribution from deep fluids, coincides with the locus of intense seismic deformation^29^, and the new springs that emerged after the Kumamoto earthquake suggest that seismicity facilitated the release of these fluids. The Aso-Kumamoto area, which is crossed by a major tectonic line including the Futagawa-Hinagu fault systems^35,36^, has repeatedly been hit by historically large crustal earthquakes (>M_w_ 6.0), and this area has been exposed to continued extensional stress over geological time^33^. The surface ruptures after the 2016 Kumamoto earthquake in the northwestern plain of the caldera^27–29^ are located in the prolongation of these fault systems (Fig. 1b). In these structural regimes, similar crustal ruptures, including minor crustal deformations, occur with each seismic event (Fig. 1b). Indeed, evidence from rupturing marks in sedimentary deposits with known stratigraphical age in this part of the caldera^28^ suggests that co-seismic deformation similar to that during the Kumamoto earthquake occurred repeatedly in this region, at least over the past 2,000 years. Therefore, water pathways similar to those observed after the 2016 event might have been formed during previous large earthquakes.

Spring water similar in composition to that emerging after the Kumamoto earthquake is also found at Kayahara (Figs 1a, 2c, and Table 1) and in several other tiny streams of high salinity water that were seeping even before the 2016 event. This observation implies that the structures facilitating discharge of the highly saline groundwaters may have been seismically initiated but are capable of maintaining long-term pathways for fluid escape. Based on our field observations, both the new spring and the previously recognized Kayahara spring have remained flowing since April 2016 and do not show any sign of weakening almost two years after the earthquake. This might continue in the future, unless clogs form within open rupture spaces and block the water pathways.

The surface ruptures observed in the northwestern plain after the Kumamoto earthquake reflect shallow deformation in the sedimentary pile^28^, extending deep enough to facilitate post-seismic escape of confined groundwater^29^. Although there is no evidence that this region of the caldera experienced motion on a fault system at greater depth in the crust^28^, the presence of the observed surface ruptures within an extensional tectonic setting is expected to favor pathways for hydrothermal fluids to ascend from the deep crust towards the surface, where they mix with infiltrating meteoric waters to form the highly concentrated confined groundwater (Fig. 3). The occurrence of deep hydrothermal fluid emergence, including the new artesian springs triggered by the 2016 Kumamoto earthquake, in the same location as intense seismic activity suggests a long-term connection between active deformation and surface water chemistry (compare locations of reported structural deformation and past earthquakes in Fig. 1a and b with sulfate rich waters labeled in red in Fig. 1c and d). This leads to the hypothesis that seismically-induced release of deep fluids in an extensional tectonic setting affects the regional surface water system and thus weathering fluxes over the timescales of repeated earthquake events.

## Deep fluid discharge fluxes

**Figure 4:**
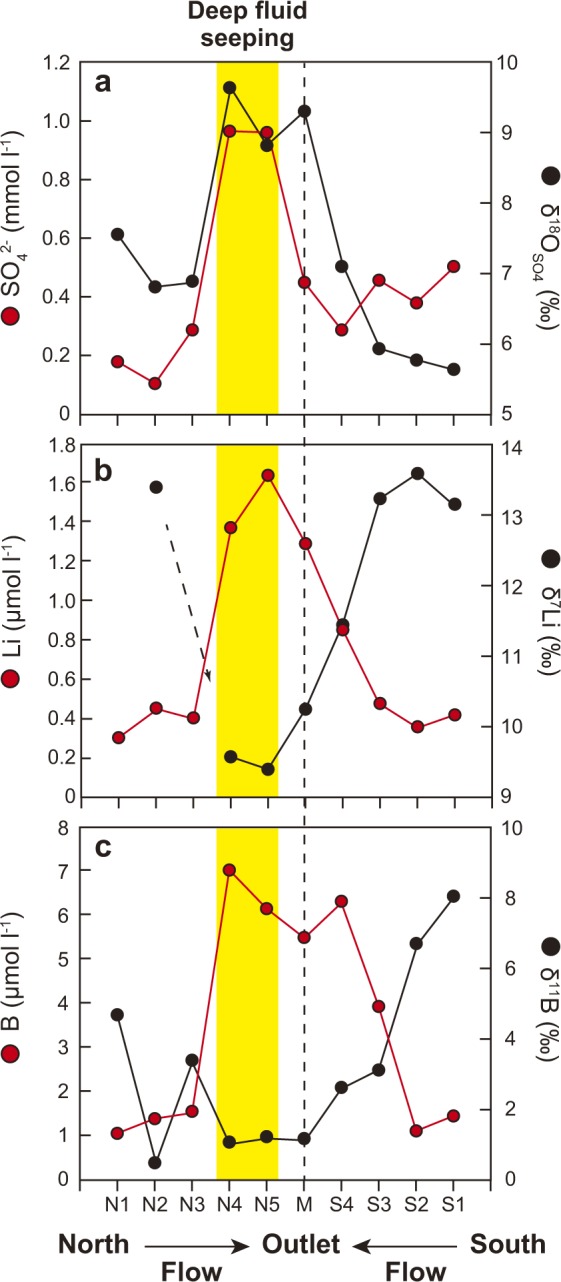
Impact of deep fluid seeping on Aso caldera’s hydrochemistry. (**a**) δ^18^O_SO4_ and [SO_4_^2−^], (**b**) δ^7^Li and [Li], and (**c**) δ^11^B and [B] evolution along the flow-path of the northern (N1 to N5) and southern (S1 to S4) Aso rivers and at the outlet of the caldera (M). See Fig. 1a for the sampling locations. The yellow band between sample locations N4 and N5 represents the northeastern area of the caldera where seeping of deep fluids occurs.

The importance of the seismically-induced solute fluxes from the northwestern plain, and the related influence of long-term release of deep fluids on lateral fluxes from the caldera, is documented by the hydrochemical contrasts between springs in this area, versus rivers upstream and downstream. Changes in sulfate concentrations along the northern main river are particularly pronounced (historical data from 1968–1995 in Figs 1 and 3a): from ca. 0.1 to 0.3 mmol l^−1^ in the upstream part of the catchment (monitoring stations N1 to N3), sulfate concentration sharply increases to ca. 1.0 mmol l^−1^ around the monitoring stations N4 and N5 (near to the spring inputs), and then decrease (ca. 0.5 mmol l^−1^) towards the outlet of the caldera. Multi-isotope data from 2014–2015 sampling campaigns allow for additional fingerprinting of the seeping deep fluids (Fig. 4). Elevated δ^18^O_SO4_ traces deep fluid contribution in the surface river water system (Fig. 4a) more efficiently than δ^34^S_SO4_, which is sensitive to redox processes such as pyrite oxidation^37,38^. δ^18^O_SO4_ in the northern river increases together with sulfate concentration, consistent with a deep groundwater source of the sulfate. Moreover, it is clear from the δ^18^O_SO4_ that the solute flux from the deep fluid seeping area leaves an imprint on the caldera outlet (station M, Ca-SO_4_ type signature), where a high value of 9.3‰ was recorded.

Isotope ratios of dissolved Li and B in the hydrothermal springs in the central part, and especially in the highly saline springs of the northwestern plain, were among the lowest of all studied waters in the caldera (2.6‰ for δ^7^Li and 1.4 to 2.6‰ for δ^11^B, respectively, Table 1). These values are consistent with the global pattern of δ^7^Li and δ^11^B in volcanic hydrothermal waters^9–11,15^. A strong impact of deep fluid seeping in the northwestern area of the caldera is also evidenced by increasing lithium and boron concentrations in tandem with decreasing δ^7^Li and δ^11^B along the northern Aso river flow-path (Fig. 4b,c). River waters flowing through the northwestern plain area mix with these deep fluid spring waters and transfer their isotopic signature downstream, to the outlet of the caldera. A similar effect of hydrothermal water inputs, influenced by the central magma sources and by the southwestern geothermal field (Fig. 1a location “e”), is corroborated by boron concentration changes around monitoring stations S3 and S4 (Fig. 4c), although this relationship is much weaker for lithium concentrations, δ^7^Li, and δ^11^B. Despite mixing with waters from sources throughout the caldera, river waters at the outlet of the system preserve the isotopic signature of deep fluids as reflected in the low δ^7^Li and δ^11^B.

To estimate the quantitative contribution of the deep fluids to the river fluxes from the caldera, a binary hydrochemical mixing model was applied using river data from upstream and downstream of the area of enhanced deep fluid discharge (see Methods and Supplementary Table 3). Applying the relatively conservative sulfate concentrations as a discrimination variable, about 3.2 to 4.1% of the water flux at the caldera outlet derives from the deep fluid seeping area. The mixing proportion varies according to the element concentration or isotopic ratio used (Supplementary Table 3), but regardless of which tracer is used, it is estimated that likely not more than 6% of the river water derives from deeper sources. Although the contribution to the water flux is small, the high solute concentrations in the deep fluids mean that these supply a substantial proportion of the total dissolved element fluxes at the caldera outlet (Supplementary Table 4): SO_4_^2−^ (56%) > Mg^2+^ (42%), Cl^−^ (41%), Na^+^ (40%) > K^+^ (26%), and Ca^2+^ (24%). Except for SiO_2_, HCO_3_^−^, and NO_3_^−^, the deep saline waters discharging in the northwestern plain deliver the majority of major dissolved ions to the river outlet (station M) even though their share of the water flux is <6% (Supplementary Table 3). The substantial contribution of the deeply-sourced spring waters to solute fluxes at the caldera outlet provides evidence for the impact of hydrothermal deep fluids on the regional hydrochemistry of an active volcanic system. We lack information about discharge rates of individual springs to confirm whether the total amount of deep fluid contribution increased after the earthquake, or hydrochemical data from the caldera outlet after the Kumamoto earthquake to assess whether there was an increase in total solute flux out of the system following this event. However, the deep fluids that are the dominant solute sources in the caldera emerge in an area of continuous seismic-induced deformation (Fig. 1, as discussed above), suggesting a seismotectonic influence on their release and thus on the overall solute budget of this system.

## Extent and implications of volcanic sulfur mediated weathering

A notable feature of the fluid chemistry of the deeply-sourced springs in the Aso caldera is the dominance of sulfate in the anion budget, accounting for 84% of the anion charge in northwestern plain springs (Table 1) and 53% in river water at the outlet of the caldera (n = 20, 1977–1995; Supplementary Table 1d). The annual average dissolved sulfur flux from the caldera watershed is estimated to be 1.7 × 10^4^ t S y^−1^ based on runoff weighted fluxes during 1981–1995 (http://www1.river.go.jp/). This dissolved flux is 4 to 36% of the annual average SO_2_ flux emitted out of the Aso volcano between 2007 and 2017 (4.6 × 10^4^ to 3.9 × 10^5^ t S y^−1^; http://www.data.jma.go.jp/svd/vois/data/fukuoka/rovdm/Asosan_rovdm/gas/gas.html), showing the relevance of hydrothermal sulfur fluxes at the scale of this volcanic system.

Stable isotopes in dissolved sulfate help to shed additional light on sulfur sources and transformations^39–41^. The S isotope composition of the sulfate from stream and spring samples (acidic stream and geothermal water in Table 1) located at the southeastern and northeastern side of the central caldera volcano exhibit δ^34^S_SO4_ of 7.2‰ and 2.6‰ (Fig. 1a and Table 1), respectively, within the typical ranges of volcanic rocks in Aso^42^, sulfate minerals and geothermal waters in some volcanic fields^12,41,43^, and volcano-hydrothermal fluids globally^44^. In contrast, the highly saline springs in the northwestern plain, including the Kayahara spring that pre-dated the 2016 earthquake and the spring we sampled that appeared after, are characterized by clearly higher δ^34^S_SO4_ (10.5‰ and 17.5‰) and δ^18^O_SO4_ (9.8‰ and 13.5‰), respectively (Fig. 1a and Table 1), with low dissolved oxygen (<2.0 mg l^−1^) and negative oxidation potential values. The high δ^34^S_SO4_ and δ^18^O_SO4_ values indicate the possibilities of either incorporation of water from a reducing environment where sulfate reduction and/or disproportionation can occur in a deep aquifer^41,45,46^, or sourcing of sulfate from disproportionation of magma derived SO_2_ in hydrothermal fluids^47^.

Other acids of magmatic origin, such as hydrochloric and carbonic acids, could also be important solute sources in the caldera^7,8^. The co-variation of SO_4_^2−^ and Cl^−^ concentrations in hydrothermal fluids (Supplementary Table 1a) is a first order indication of the importance of hydrochloric acid, but Cl^−^ has an average charge equivalent ratio relative to sulfate of only 1/5 in the Aso spring waters. The magmatic CO_2_ contribution previously identified for the Aso caldera watershed^8^ seems proportionally negligible in the northwestern plain area, based on the low δ^13^C_DIC_ compositions of −11.3‰ to −10.9‰ at the river water sampling stations N4 and N5 relative to typical magmatic value of ca. −5‰ within Aso caldera watershed^8^ (though degassing may induce isotopic fractionation of river water DIC), and on the very low alkalinity contribution to the ion charge balance among major dissolved anions for spring- and ground-waters (Supplementary Table 2 and Fig. 1c). Thus, while magmatic CO_2_ may be important elsewhere in the Aso system, it appears to have a minor impact on geochemical fluxes from the northwestern plain, when compared to the sulfur contributions. NO_3_^−^ concentrations, part of which are of anthropogenic origin^45,48^, were under detection limit (<0.001 mmol l^−1^) for the highly saline spring water and were also very low for river systems in the Aso watershed (Supplementary Table 2).

The anion sources have important implications for the overall output of dissolved elements that leave the caldera catchment, and for inferences concerning the carbon cycle. According to the anion-cation charge balance, and because dissolved sulfate is predominantly of magmatic origin, more than half of the total cation equivalents delivered at the outlet of the caldera might be attributed to magmatic S-mediated weathering. Thus, we conclude that the primary driver of weathering and solute generation in this region is associated with magmatically-derived sulfur rather than CO_2_. Weathering driven by CO_2_ that is derived from the atmosphere and cycled through the ecosystem, for example in soils or shallow groundwaters, produces net alkalinity that effectively draws down atmospheric CO_2_^49,50^. Similarly, rock alteration with magmatic CO_2_ sources neutralizes this CO_2_, inhibiting its release to the atmosphere. In contrast, the volcanic S-mediated weathering that dominates in the Aso caldera system is not directly associated with CO_2_ sequestration. Thus, the apparent high weathering rates inferred for this system from solute fluxes may not directly represent the extent of CO_2_ consumption.

## Conclusions and implications for global weathering fluxes

Based on historical data from the Aso caldera river water at the outlet, the discharge weighted average total dissolved solid load (TDS) is 235 mg l^−1^ resulting in a total solute yield of 458 t km^−2^ y^−1^ (Supplementary Table 1d). Similarly, the yield of dissolved Na^+^  + K^+^  + Ca^2+^  + Mg^2+^  + SiO_2_ from the caldera is estimated to be 205 t km^−2^ y^−1^. These values are higher than those reported for many active volcanic areas^7,51,52^ and comparable to regions with the world’s highest weathering fluxes^53–56^. We have shown that a majority of the solute flux from the Aso caldera derives from deep fluids that discharge from groundwater aquifers in the northwestern plain, despite its small area (approximately 25 km^2^) that comprises less than 7% of the total catchment area of 380 km^2^. The release of these fluids, and the resulting long-lived hydrochemical anomaly in this region, is associated with the surface rupture system crossing this part of the caldera. The springs that emerged after the Kumamoto earthquake reveal how seismic activity along the tectonic line can open up pathways for fluid escape from the deep subsurface, illustrating how the seismotectonics of this region contribute to the high total solute flux out of the caldera system.

Long-lived hydrochemical anomalies have been documented around the world in active volcanic areas (e.g., Kawah Ijen, Indonesia; Poás volcano, Costa Rica; and Copahue volcano, Argentina), with riverine fluxes of solutes in these locations predominantly derived from point sources in calderas even without seismically facilitated mobilization of deeply derived fluid^57–60^. Nonetheless, we propose that seismotectonically-enhanced local anomalies might exist in some settings comparable to Aso, and might, as shown here, significantly increase the dissolved load fluxes delivered from the land surface to the oceans. If such settings could be mapped globally, and their relative contribution to elevated fluxes described by scaling laws, the global relevance of seismically-mobilized deep fluid sources of dissolved elements to the ocean could be estimated. The present study demonstrates that earthquakes can open preferential flow-paths capable of releasing deep hydrothermal fluids in active volcanic areas, and we suggest that this effect might explain in part the widely recognized disproportionate contribution of active volcanic areas to weathering fluxes at the global scale^1–5^. The extent to which these deep fluids are associated with S-mediated versus CO_2_-mediated weathering reactions will have important implications for the role of these volcanic areas in the global carbon cycle.

## Methods

Methods, including statements of data availability and any associated accession codes and references, are available as supplementary material.

### Outline of the study area

The Aso caldera is situated within the Oita-Kumamoto Tectonic Line^35,61,62^, one of the major tectonic lines in Japan with NE-SW orientation, consisting of the three active strike-slip fault systems Hinagu, Futagawa, and Imahata. The Aso caldera watershed is on the eastern extension of the Hinagu-Futagawa fault systems (Fig. 1). Tectonically, this area has been subject to an extensional stress field^63–65^. The average horizontal slip rate for the fault system has been estimated to be 0.88 mm y^−1^ over the late Quaternary period^36^.

In 2016 the destructive inland Kumamoto earthquake started by a large foreshock of M_w_ 6.2 on April 14^th^, followed by the main shock of M_w_ 7.0 on April 16^th^ (http://www.jma.go.jp/jma/en/2016_Kumamoto_Earthquake/2016_Kumamoto_Earthquake.html). It was the largest earthquake in Kyushu induced by the Hinagu-Futagawa faults activities since 1885, when observation records started^33^. The active Hinagu-Futagawa faults system is located in the southwest of the Aso caldera and extends eastwards directly through the northwestern part of Aso caldera, in the northern river “Kurokawa” catchment (Fig. 1). The surface ruptures and land slips happened in the northwestern plain area, along this tectonic line^29,66–71^. No reverse faults have been observed, and all fault ruptures confirmed in the field are strike-slip or normal faults^33^.

The surface geology of the study area consists of Quaternary volcanic rocks^72–79^ of various compositions, from basalts to rhyolites^72,75,79–84^, and sedimentary deposits. A total of 27 volcanic cones are recognized in the caldera central mountains, of which Naka-dake is the only active crater where volcanic gas monitoring station is installed (http://www.jma.go.jp/jma/indexe.html, http://www.data.jma.go.jp/svd/vois/data/fukuoka/rovdm/Asosan_rovdm/gas/gas.html).

The Aso caldera watershed (380 km^2^) is developed within a large caldera topology (25 km × 18 km). The ring-shape caldera somma ridge (highest peak: 1,154 m) forms the watershed divide. The central volcanic mountains (highest peak: 1,592 m) are situated in the central part of the caldera (Fig. 1) and active volcanic fumaroles (Naka-dake) are present in the central crater. Both caldera somma and central mountains are the main water recharge areas of the watershed. The watershed can be divided into two sub-watershed systems: the Kurokawa (means “black river” in Japanese) catchment in the north and the Shirakawa (means “white river”) catchment in the south. These two rivers flow westward along a topographical gradient, meeting each other at the western endpoint, and flow out of the caldera immediately after the confluence (Fig. 1). River discharge rates have been monitored at two gauging stations, one on the downstream reaches of the Shirakawa river (southern river) and the other at the caldera outlet just after Shirakawa and Kurokawa (northern river) confluence (river sampling station M, http://www1.river.go.jp/), with average annual (1996–2016) water fluxes of 0.34 and 0.74 km^3^ y^−1^, respectively.

The climate of the study area is categorized as warm and humid, influenced by the Asian monsoon and shows four distinct seasons (http://www.data.jma.go.jp/gmd/cpd/longfcst/en/tourist.html). The annual average precipitation in the Aso area is 2,832 mm y^−1^ with an average temperature of 12.9 °C (1981–2010; https://weather.time-j.net/Climate/Chart/asootohime). The rainy season (June – July) accounts for ~40% of the total annual precipitation.

### Historical hydrochemistry data sources

For the basic hydrochemistry within the watershed (Fig. 1c,d) we used major ion (Na^+^, K^+^, Ca^2+^, Mg^2+^, Cl^−^, HCO_3_^−^, SO_4_^2−^, and NO_3_^−^) concentration data published in a Japanese article^85^ and reports of the Bunsei University^86,87^, which are the most comprehensive archives, geographically and temporally, for this area (Supplementary Table 1 and Supplementary Fig. 1 for its location). Average concentrations were used to illustrate the general hydrochemistry in a Stiff diagram style in Fig. 1c and Fig. 1d maps^88^. For spring- and ground-waters (Fig. 1c) we combined two datasets: one is a single sampling survey during 1968–1969 (ref.^85^; Supplementary Table 1a) and the other is from multiple sample monitoring between 1977 and 1995, with up to 20 measurements at the same location in different seasons (Supplementary Table 1b,c)^86^. For the river water data, we used a database of multiple samples collected between 1977 and 1995 (ref.^87^; Supplementary Table 1d). The documented hydrochemical type of water (e.g., classification as Ca-HCO_3_ or Ca-SO_4_) as well as elemental ratios are relatively invariant with time in the datasets, but concentrations change with discharge, due to rainwater dilution effects, especially for the river water samples^87^.

### New data sources

Concentrations and isotope data for river (in Fig. 4) and hydrothermal waters (in Table 1) are from samples collected during five sampling campaigns in different seasons: Mar-April 2014, September-October 2014, May 2015, June-July 2015, and July 2016. At some sites, samples were not collected during all of the field campaigns. Some seasonal changes in hydrochemistry and isotope ratios are expected due to rainwater dilution, but the source characteristics and the type of hydrochemistry along the river flow path remained the same regardless of the sampling season, as confirmed from the longer time series datasets (see above). Therefore, for acid streams and geothermal water, average values are given in Table 1 and plotted in Fig. 4, and all measured values are provided in Supplementary Table 2. Detailed spatiotemporal changes in isotope compositions will be discussed in separate papers. The samples from three volcanic-hydrothermal fluids (Table 1) were each collected once after the main shock of the 2016 Kumamoto earthquake (16 April 2016), on 3 July 2016 (highly saline fluid that emerged after the quake and hot spring “Uchinomaki”) and on 28 July 2016 (highly saline fluid that also existed before the quake, “Kayahara”). For these samples, the values provided in Table 1 are those measured at these single sampling times.

### Binary mixing calculations

Using equations (1) and (2) below, we calculated the mass fraction of deep fluid (*f*) in the river draining the northwestern plain area, based on the concentrations and isotope ratios of selected dissolved species (*E*) in the two mixing components, deep fluid and upstream river water from stations N2 and N3:

1 $${E}_{X}={E}_{D}\cdot f+{E}_{US}\cdot (1-f)$$

and

2 $$\delta {E}_{X}=\delta {E}_{D}\cdot ({E}_{D}/{E}_{X})\cdot f+\delta {E}_{US}\cdot ({E}_{US}/{E}_{X})\cdot (1\mbox{--}f)$$

where *E*_*X*_, *E*_*D*_, *E*_*US*_, δ*E*_*X*_, δ*E*_*D*_, and δ*E*_*US*_ are the concentrations and isotope ratios for mixed river water (*X*), deep fluid (*D*), and upstream river water (*US*), respectively. *f* is a variable parameter, identified from the best fit between calculated *E*_*X*_ and δ*E*_*X*_ and observed concentrations and isotope ratios for downstream river water from stations N4 and N5 (Supplementary Table 3).

### Analytical procedures

Water samples were filtered in the field through cellulose acetate filters at 0.2 or 0.45 μm pore diameter, stored in polypropylene bottles, and preserved in dark and cooled containers at a temperature around 5 °C. Samples were acidified to pH 2 with ultra-pure nitric acid, except for the vials dedicated to anion analyses. The alkalinity was measured by automated Metrohm titration system. The Na^+^, K^+^, Ca^2+^, Mg^2+^, Cl^−^, SO_4_^2−^, and NO_3_^−^ concentrations were analyzed by ion chromatography at Hamburg University and Kumamoto University (Compact IC 761, Metrohm, Switzerland). The Li^+^ and B concentrations were analyzed by ICP-MS in Kumamoto University (NexION 300, Perkin-Elmer Co., Ltd, USA) and Institut de Physique du Globe de Paris (IPGP) (Agilent7900, Agilent Technologies, USA). Hydrogen and oxygen isotope ratios of water were determined by Laser-CRDS using a Picarro L2140i at the Leibniz IOW^89^ or continuous-flow gas-ratio mass spectrometer at the Kumamoto University (Delta V Advantage, Thermo Fisher Scientific, USA). The analytical precision of the water isotope measurements was better than < ±0.05‰ and < ±0.5‰, respectively. The carbon isotope ratios of dissolved inorganic carbon were analyzed at the Leibniz IOW using a Thermo Gasbench II coupled to a Thermo Finnigan MAT 253 gas mass spectrometer via a Thermo Conflo IV split interface as reported previously^90^, with a precision better than ± 0.1‰. The stable sulfur isotope ratio of sulfate was determined by continuous-flow gas isotope-ratio mass spectrometry (Delta V Advantage, Thermo Fisher Scientific, USA) coupled with an elemental analyzer (Flash 2000, Thermo Fisher Scientific, USA) at the Kumamoto University, following the methods previously developed^46,91^. The analytical precision was better than ± 0.2‰ during the analytical session. The oxygen stable isotope ratio of sulfate was determined by the same mass spectrometer (Delta V Advantage, Thermo Fisher Scientific, USA) coupled with a high temperature conversion elemental analyzer (TC/EA, Thermo Fisher Scientific, USA) at the Kumamoto University. The precision was estimated to be better than ± 0.3‰ based on repeated measurement of working standards. Lithium isotopic analysis was performed by MC-ICP-MS (Neptune, Thermo Scientific, USA) at Caltech, with analytical precision ± 0.80‰ (ref.^92^). Boron isotope ratios were determined by MC-ICP-MS (Neptune, Thermo Scientific, USA) at IPGP with analytical precision of < ±0.25‰ (refs^93,94^). All isotope ratios were expressed in delta-notation (δ) in per mill unit (‰) with respect to international standards: Vienna Standard Mean Ocean Water for δD and δ^18^O, L-SVEC for δ^7^Li, NIST SRM 951 for δ^11^B, Vienna Peedee Belemnite for δ^13^C, and Vienna Canyon Diablo Troilite for δ^34^S, respectively.

## Electronic supplementary material

Supplementary Information

Supplementary Table 1a-d

Supplementary Table 2

Supplementary Table 3

Supplementary Table 4
