# Supplementary material for: Earthquake-induced structural deformations enhance long-term solute fluxes from active volcanic systems
Source: Sci Rep. 2018 Oct 4;8:14809. doi: 10.1038/s41598-018-32735-1 (PMC6172214; doi:10.1038/s41598-018-32735-1)
Supplement: Supplementary file 1 — Supplementary Information [file 41598_2018_32735_MOESM1_ESM.docx]

**Supplementary Information**

**Airticle in *Scientific Reports***

**Earthquake-induced structural deformations enhance long-term solute fluxes from active volcanic systems**

Takahiro Hosono^1,2,3^*, Jens Hartmann^1,4^, Pascale Louvat^3^, Thorben Amann^4^, Kirstin E. Washington^5^, A. Joshua West^5^, Koki Okamura^2^, Michael E. Böttcher^6^, and Jérôme Gaillardet^3^

^1^Priority Organization for Innovation and Excellence, Kumamoto University, 2-39-1 Kurokami, Kumamoto 860-8555, Japan

^2^Department of Earth Science, Kumamoto University, 2-39-1 Kurokami, Kumamoto 860-8555, Japan

^3^Institut de Physique du Globe de Paris, Sorbonne Paris Cité, Univ Paris Diderot, UMR 7154 CNRS, Paris, France

^4^Institute for Geology, Universität Hamburg, Center for Earth System Research and Sustainability (CEN), Bundesstrasse 55, 20146 Hamburg, Germany

^5^Department of Earth Sciences, University of Southern California, 3651 Trousdale Parkway, Los Angeles, CA 90089, USA

^6^Geochemistry & Isotope Biogeochemistry Group, Leibniz Institute for Baltic Sea Research (IOW), Seestrasse 15, D-18119 Warnemünde, Germany

*Corresponding author: hosono@kumamoto-u.ac.jp

**Supplementary Figure**


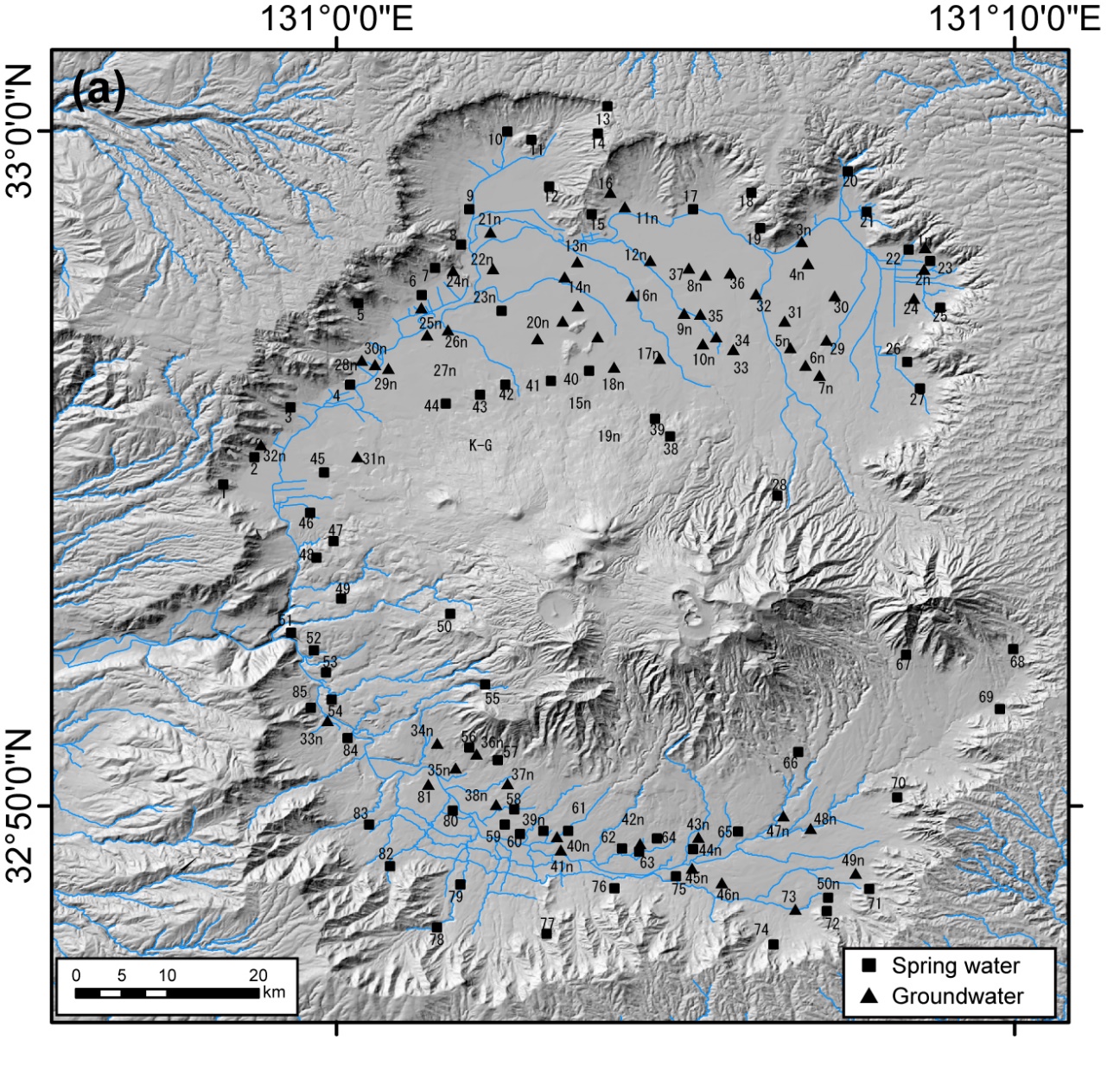


**
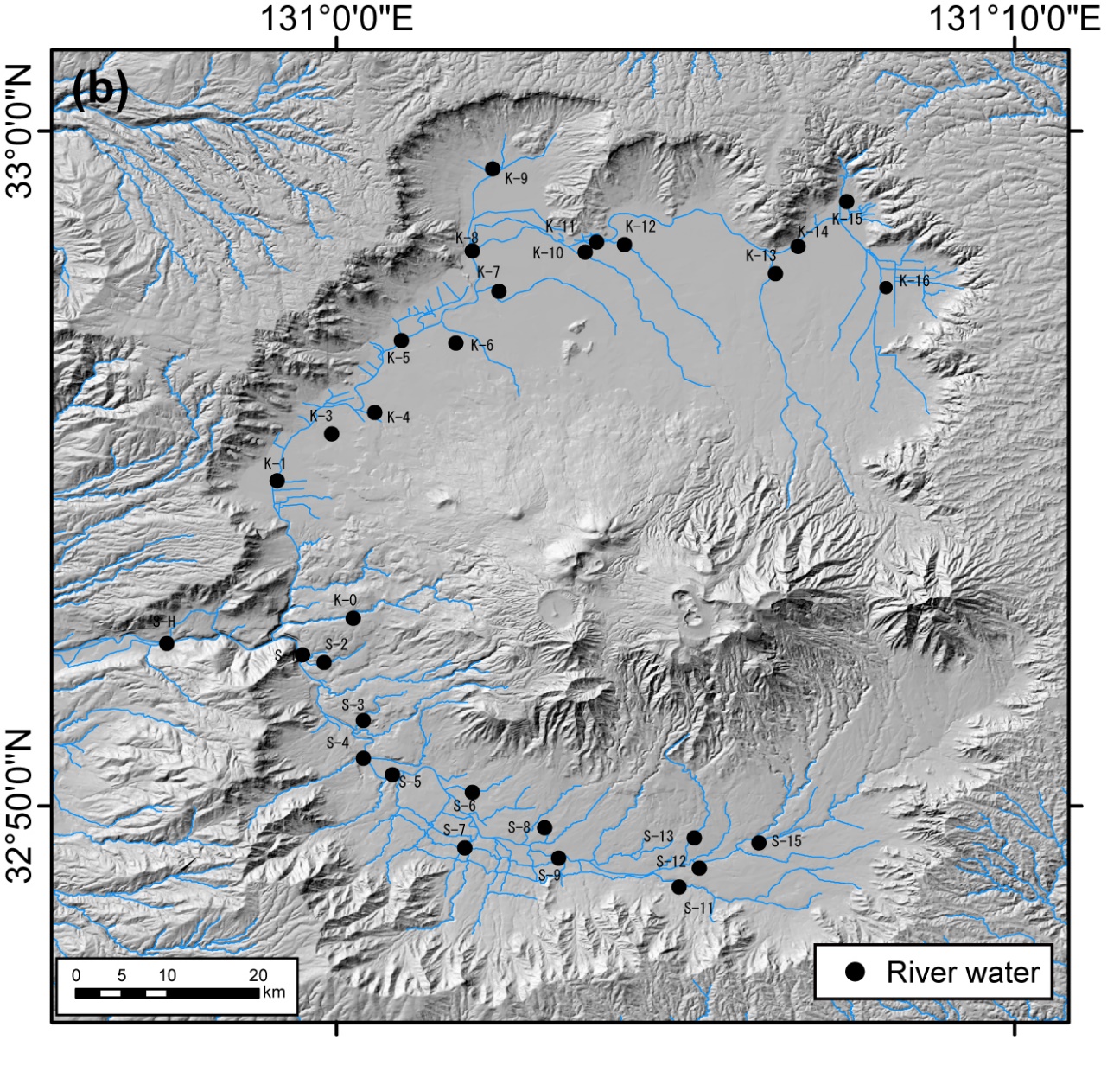
Supplementary Figure 1. Sampling location for the historical hydrochemical data.** Location numbers (see Supplementary Table 1) are labeled at each sampling point and shown for (a) spring water and groundwater^85,86^ and (b) river water^87^, respectively. The map was illustrated by using ArcGIS Desktop (Esri).

**Supplementary Table**

**Supplementary Table 1a: Historical data sources for the basic hydrochemistry within the Aso watershed for single groundwater sampling survey during 1968-1969 after Nagai et al. (1989) (ref. 85).** (see Excel file)

**Supplementary Table 1b: Historical data sources for the basic hydrochemistry within the Aso watershed for repeated groundwater sampling survey during 1977-1995 after Shimano (1997) (ref. 86).** (see Excel file)

**Supplementary Table 1c: Historical data sources for the basic hydrochemistry within the Aso watershed for repeated groundwater sampling (average) survey during 1977-1995 after Shimano (1997) (ref. 87).** (see Excel file)

**Supplementary Table 1d: Historical data sources for the basic hydrochemistry within the Aso watershed for repeated river water sampling survey during 1977-1995 after Shimano (1999) (ref. 87).** (see Excel file)

**Supplementary Table 2: Analytical results for hydrochemistry and isotope ratios for water collected from Aso caldera watershed.** (see Excel file)

**Supplementary Table 3: Proportions (%) of water from highly saline deep fluids (binary mixing model, see Methods).** (see Excel file)

**Supplementary Table 4: Proportions of each chemical flux among the total flux from caldera outlet through northern river relative to southern river.** (see Excel file)
